# 2-Morpholino-4-oxo-4,5-dihydro­thio­phene-3-carbonitrile

**DOI:** 10.1107/S1600536809041737

**Published:** 2009-10-17

**Authors:** JinJiang Zhu, Kevin K. Liu, Matthew A. Marx, Arnold L. Rheingold, Alex Yanovsky

**Affiliations:** a Pfizer Global Research and Development, La Jolla Labs, 10770 Science Center Drive, San Diego, CA 92121, USA; b Department of Chemistry and Biochemistry, University of California, San Diego, 9500 Gilman Drive, La Jolla, CA 92093, USA

## Abstract

The title compound, C_9_H_10_N_2_O_2_S, was obtained from the treatment of ethyl 4-cyano-3-hydr­oxy-5-morpholinothio­phene-2-carboxyl­ate with concentrated HCl. The mean plane of the essentially planar dihydro­thio­phene ring is almost orthogonal to the mirror plane of the *N*-morpholine substituent, making a dihedral angle of 87.2 (2)°.

## Related literature

For the structure of a similar compound with the morpholine substituent attached to dihydro­thio­phene ring, see: Moghaddam *et al.* (2005 ▶).
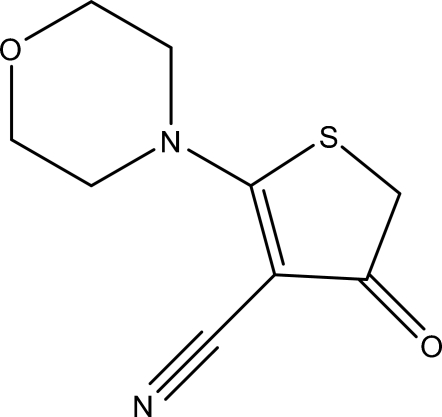


## Experimental

### 

#### Crystal data

C_9_H_10_N_2_O_2_S
*M* *_r_* = 210.25
Monoclinic, 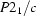

*a* = 7.1931 (3) Å
*b* = 17.3275 (8) Å
*c* = 7.2793 (3) Å
β = 94.506 (2)°
*V* = 904.48 (7) Å^3^
*Z* = 4
Cu *K*α radiation
μ = 2.98 mm^−1^
*T* = 100 K
0.41 × 0.20 × 0.08 mm

#### Data collection

Bruker Kappa APEXII diffractometer
Absorption correction: multi-scan (*SADABS*; Bruker, 2001 ▶) *T*_min_ = 0.765, *T*_max_ = 0.919
7337 measured reflections
1607 independent reflections
1531 reflections with *I* > 2σ(*I*)
*R*_int_ = 0.027

#### Refinement

*R*[*F* ^2^ > 2σ(*F* ^2^)] = 0.026
*wR*(*F* ^2^) = 0.068
*S* = 1.08
1607 reflections
128 parameters
H-atom parameters constrained
Δρ_max_ = 0.29 e Å^−3^
Δρ_min_ = −0.20 e Å^−3^

### 

Data collection: *APEX2* (Bruker, 2007 ▶); cell refinement: *SAINT* (Bruker, 2007 ▶); data reduction: *SAINT*; program(s) used to solve structure: *SHELXS97* (Sheldrick, 2008 ▶); program(s) used to refine structure: *SHELXL97* (Sheldrick, 2008 ▶); molecular graphics: *SHELXTL* (Sheldrick, 2008 ▶); software used to prepare material for publication: *SHELXTL*.

## Supplementary Material

Crystal structure: contains datablocks pflj124, I. DOI: 10.1107/S1600536809041737/dn2498sup1.cif

Structure factors: contains datablocks I. DOI: 10.1107/S1600536809041737/dn2498Isup2.hkl

Additional supplementary materials:  crystallographic information; 3D view; checkCIF report

## Figures and Tables

**Fig. 1.**
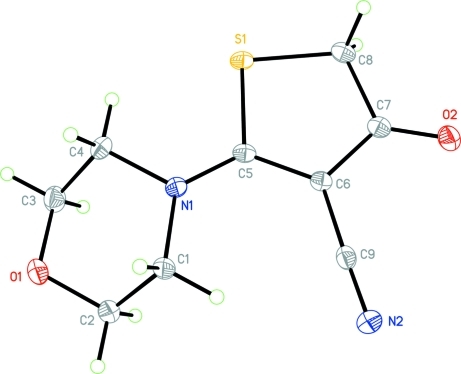
Molecular structure of the title compound, showing 50% probability displacement ellipsoids and atom numbering scheme. H atoms are drawn as circles with arbitrary small radius.

## supplementary crystallographic information

### Comment

The title compound was obtained *via* the treatment of ethyl 4-cyano-3-hydroxy-5-morpholinothiophene-2-carboxylate with concentrated HCl, and its structural formula was confirmed by the present study (Fig. 1).

Dihydrothiophene ring C5/C6/C7/C8/S1 is planar within 0.02 Å. Its least squares plane is almost orthogonal to the mirror plane of the *N*-morpholine substituent passing through C5, N1 and O1 atoms: the corresponding dihedral angle being 92.8 (2)°. Similar conformation is observed in the related structure with morpholine substituent attached to dihydrothiophene ring (Moghaddam *et al.*, 2005).

### Experimental

Into a suspension of ethyl 4-cyano-3-hydroxy-5-morpholinothiophene-2-carboxylate (100 mg, 0.35 mmol) in MeOH (1.2 ml), was added concentrated HCl (0.2 ml) with stirring. The reaction mixture was heated in an oil bath at 60°C for 48 h to form a clear solution. The reaction solution was cooled to room temperature and the solvent was removed under reduced pressure. The resulting residue was neutralized with 2 N NaOH to pH 4. The precipitate was collected by filtration and rinsed with a solution of water/MeOH. The sample was dried under high vacuum to afford the desired compound as a white solid (52.1 mg, 58% yield). LC—MS (APCI, *M*+1) 211.2; ^1^H NMR (300 MHz, DMSO-d_6_) δ p.p.m. 3.87 (s, 3 H), 3.84 (dd, J=5.84, 2.07 Hz, 2 H), 3.68 - 3.79 (m, 5 H). The product was recrystallized from EtOAc/hexane/dichloromethane to yield single crystals suitable for X-ray diffraction studies.

### Refinement

All H atoms were placed in geometrically calculated positions (C—H 0.99 Å) and included in the refinement in riding motion approximation. The *U*_iso_(H) were set to 1.2*U*_eq_ of the carrying atom.

### Crystal data

**Table:**

C_9_H_10_N_2_O_2_S	*F*(000) = 440
*M**_r_* = 210.25	*D*_x_ = 1.544 Mg m^−^^3^
Monoclinic, *P*2_1_/*c*	Cu *K*α radiation, λ = 1.54178 Å
Hall symbol: -P 2ybc	Cell parameters from 2017 reflections
*a* = 7.1931 (3) Å	θ = 8.0–49.4°
*b* = 17.3275 (8) Å	µ = 2.98 mm^−^^1^
*c* = 7.2793 (3) Å	*T* = 100 K
β = 94.506 (2)°	Blade, colorless
*V* = 904.48 (7) Å^3^	0.41 × 0.20 × 0.08 mm
*Z* = 4	

### Data collection

**Table:**

Bruker Kappa APEXII diffractometer	1607 independent reflections
Radiation source: fine-focus sealed tube	1531 reflections with *I* > 2σ(*I*)
graphite	*R*_int_ = 0.027
phi and ω scans	θ_max_ = 68.3°, θ_min_ = 5.1°
Absorption correction: multi-scan (*SADABS*; Bruker, 2001)	*h* = −7→8
*T*_min_ = 0.765, *T*_max_ = 0.919	*k* = −20→20
7337 measured reflections	*l* = −6→8

### Refinement

**Table:**

Refinement on *F*^2^	Secondary atom site location: difference Fourier map
Least-squares matrix: full	Hydrogen site location: inferred from neighbouring sites
*R*[*F*^2^ > 2σ(*F*^2^)] = 0.026	H-atom parameters constrained
*wR*(*F*^2^) = 0.068	*w* = 1/[σ^2^(*F*_o_^2^) + (0.0333*P*)^2^ + 0.4*P*] where *P* = (*F*_o_^2^ + 2*F*_c_^2^)/3
*S* = 1.08	(Δ/σ)_max_ = 0.005
1607 reflections	Δρ_max_ = 0.29 e Å^−^^3^
128 parameters	Δρ_min_ = −0.20 e Å^−^^3^
0 restraints	Extinction correction: *SHELXL97* (Sheldrick, 2008), Fc^*^=kFc[1+0.001xFc^2^λ^3^/sin(2θ)]^-1/4^
Primary atom site location: structure-invariant direct methods	Extinction coefficient: 0.0051 (4)

### Special details

**Table:**

Geometry. All e.s.d.'s (except the e.s.d. in the dihedral angle between two l.s. planes) are estimated using the full covariance matrix. The cell e.s.d.'s are taken into account individually in the estimation of e.s.d.'s in distances, angles and torsion angles; correlations between e.s.d.'s in cell parameters are only used when they are defined by crystal symmetry. An approximate (isotropic) treatment of cell e.s.d.'s is used for estimating e.s.d.'s involving l.s. planes.
Refinement. Refinement of *F*^2^ against ALL reflections. The weighted *R*-factor *wR* and goodness of fit *S* are based on *F*^2^, conventional *R*-factors *R* are based on *F*, with *F* set to zero for negative *F*^2^. The threshold expression of *F*^2^ > σ(*F*^2^) is used only for calculating *R*-factors(gt) *etc*. and is not relevant to the choice of reflections for refinement. *R*-factors based on *F*^2^ are statistically about twice as large as those based on *F*, and *R*- factors based on ALL data will be even larger.

### Fractional atomic coordinates and isotropic or equivalent isotropic displacement parameters (Å^2^)

**Table:**

	*x*	*y*	*z*	*U*_iso_*/*U*_eq_	
S1	0.41531 (4)	0.190051 (19)	0.14477 (4)	0.01446 (13)	
O1	0.93155 (14)	−0.01397 (6)	0.25143 (14)	0.0203 (2)	
O2	0.60927 (14)	0.39685 (6)	0.22161 (14)	0.0206 (2)	
N2	1.03469 (17)	0.30903 (7)	0.41842 (18)	0.0206 (3)	
N1	0.72767 (15)	0.12491 (7)	0.28592 (16)	0.0153 (3)	
C3	0.7576 (2)	−0.00224 (8)	0.1488 (2)	0.0202 (3)	
H3A	0.6948	−0.0526	0.1261	0.024*	
H3B	0.7789	0.0210	0.0281	0.024*	
C4	0.63342 (19)	0.05034 (8)	0.2519 (2)	0.0186 (3)	
H4A	0.5133	0.0583	0.1783	0.022*	
H4B	0.6072	0.0263	0.3706	0.022*	
C5	0.64590 (18)	0.19201 (8)	0.24689 (18)	0.0127 (3)	
C6	0.71300 (18)	0.26741 (8)	0.27408 (17)	0.0132 (3)	
C7	0.58227 (19)	0.32720 (8)	0.21546 (17)	0.0143 (3)	
C9	0.89130 (19)	0.28941 (8)	0.35321 (18)	0.0147 (3)	
C8	0.39555 (18)	0.29364 (8)	0.14293 (18)	0.0163 (3)	
H8A	0.3622	0.3121	0.0159	0.020*	
H8B	0.2968	0.3101	0.2218	0.020*	
C2	1.0259 (2)	0.05782 (8)	0.2777 (2)	0.0204 (3)	
H2A	1.0490	0.0799	0.1562	0.025*	
H2B	1.1482	0.0491	0.3468	0.025*	
C1	0.91428 (19)	0.11467 (8)	0.3821 (2)	0.0191 (3)	
H1A	0.9019	0.0954	0.5086	0.023*	
H1B	0.9799	0.1649	0.3912	0.023*	

### Atomic displacement parameters (Å^2^)

**Table:**

	*U*^11^	*U*^22^	*U*^33^	*U*^12^	*U*^13^	*U*^23^
S1	0.01039 (19)	0.0182 (2)	0.0144 (2)	−0.00046 (11)	−0.00152 (13)	−0.00068 (11)
O1	0.0208 (5)	0.0133 (5)	0.0264 (5)	0.0025 (4)	−0.0004 (4)	0.0007 (4)
O2	0.0198 (5)	0.0151 (5)	0.0270 (5)	0.0026 (4)	0.0030 (4)	0.0025 (4)
N2	0.0156 (6)	0.0184 (6)	0.0273 (7)	−0.0014 (5)	−0.0015 (5)	−0.0033 (5)
N1	0.0123 (5)	0.0139 (6)	0.0191 (6)	−0.0009 (4)	−0.0025 (5)	−0.0008 (4)
C3	0.0230 (8)	0.0154 (7)	0.0216 (7)	−0.0006 (5)	−0.0021 (6)	−0.0011 (5)
C4	0.0162 (7)	0.0139 (7)	0.0253 (7)	−0.0039 (5)	−0.0008 (6)	−0.0010 (5)
C5	0.0111 (6)	0.0169 (7)	0.0102 (6)	0.0006 (5)	0.0018 (5)	−0.0011 (5)
C6	0.0118 (6)	0.0149 (7)	0.0129 (6)	0.0007 (5)	0.0014 (5)	−0.0003 (5)
C7	0.0142 (6)	0.0174 (7)	0.0118 (6)	0.0011 (5)	0.0040 (5)	0.0008 (5)
C9	0.0167 (7)	0.0116 (6)	0.0161 (6)	0.0015 (5)	0.0036 (5)	−0.0006 (5)
C8	0.0135 (7)	0.0193 (7)	0.0159 (7)	0.0031 (5)	−0.0001 (5)	0.0011 (5)
C2	0.0158 (7)	0.0160 (7)	0.0293 (8)	0.0006 (5)	0.0008 (6)	0.0049 (6)
C1	0.0147 (7)	0.0145 (7)	0.0267 (7)	0.0004 (5)	−0.0071 (6)	0.0005 (5)

### Geometric parameters (Å, °)

**Table:**

S1—C5	1.7639 (13)	C4—H4B	0.9900
S1—C8	1.8004 (14)	C5—C6	1.4014 (18)
O1—C3	1.4204 (17)	C6—C9	1.4163 (18)
O1—C2	1.4227 (17)	C6—C7	1.4410 (18)
O2—C7	1.2227 (17)	C7—C8	1.5197 (18)
N2—C9	1.1523 (19)	C8—H8A	0.9900
N1—C5	1.3238 (17)	C8—H8B	0.9900
N1—C4	1.4710 (17)	C2—C1	1.513 (2)
N1—C1	1.4751 (17)	C2—H2A	0.9900
C3—C4	1.515 (2)	C2—H2B	0.9900
C3—H3A	0.9900	C1—H1A	0.9900
C3—H3B	0.9900	C1—H1B	0.9900
C4—H4A	0.9900		
			
C5—S1—C8	93.15 (6)	O2—C7—C6	126.92 (13)
C3—O1—C2	109.68 (10)	O2—C7—C8	121.60 (12)
C5—N1—C4	122.97 (11)	C6—C7—C8	111.48 (12)
C5—N1—C1	125.44 (11)	N2—C9—C6	178.37 (15)
C4—N1—C1	111.37 (11)	C7—C8—S1	108.17 (9)
O1—C3—C4	110.79 (11)	C7—C8—H8A	110.1
O1—C3—H3A	109.5	S1—C8—H8A	110.1
C4—C3—H3A	109.5	C7—C8—H8B	110.1
O1—C3—H3B	109.5	S1—C8—H8B	110.1
C4—C3—H3B	109.5	H8A—C8—H8B	108.4
H3A—C3—H3B	108.1	O1—C2—C1	111.75 (11)
N1—C4—C3	109.24 (11)	O1—C2—H2A	109.3
N1—C4—H4A	109.8	C1—C2—H2A	109.3
C3—C4—H4A	109.8	O1—C2—H2B	109.3
N1—C4—H4B	109.8	C1—C2—H2B	109.3
C3—C4—H4B	109.8	H2A—C2—H2B	107.9
H4A—C4—H4B	108.3	N1—C1—C2	109.81 (11)
N1—C5—C6	130.26 (12)	N1—C1—H1A	109.7
N1—C5—S1	117.43 (10)	C2—C1—H1A	109.7
C6—C5—S1	112.30 (10)	N1—C1—H1B	109.7
C5—C6—C9	126.81 (12)	C2—C1—H1B	109.7
C5—C6—C7	114.77 (12)	H1A—C1—H1B	108.2
C9—C6—C7	118.42 (12)		

## References

[bb1] Bruker (2001). *SADABS* Bruker AXS Inc., Madison, Wisconsin, USA.

[bb2] Bruker (2007). *APEX2 *and *SAINT* Bruker AXS Inc., Madison, Wisconsin, USA.

[bb3] Moghaddam, F. M., Boeini, H. Z., Bagheri, M., Ruëdi, P. & Linden, A. (2005). *Sulfur Chem.* **26**, 245–250.

[bb4] Sheldrick, G. M. (2008). *Acta Cryst.* A**64**, 112–122. 10.1107/S0108767307043930 18156677

